# Poly[(μ_5_-2,2′-bipyridine-5,5′-dicarboxyl­ato)lead(II)]

**DOI:** 10.1107/S1600536812035647

**Published:** 2012-08-23

**Authors:** Mustafa Sertçelik, Nagihan Çaylak Delibaş, Sabri Çevik, Hacali Necefoğlu, Tuncer Hökelek

**Affiliations:** a Department of Chemistry, Kafkas University, 36100 Kars, Turkey; b Department of Physics, Sakarya University, 54187 Esentepe, Sakarya, Turkey; c Department of Chemistry, Afyon Kocatepe University, 03200 Afyonkarahisar, Turkey; d Department of Physics, Hacettepe University, 06800 Beytepe, Ankara, Turkey

## Abstract

In the title polymeric compound, [Pb(C_12_H_6_N_2_O_4_)]_*n*_, the Pb^II^ cation, located on a mirror plane, is *N*,*N*′-chelated by a 2-2′-bipyridine-5,5′-dicarboxyl­ate (bpdc) anion and is further coordinated by six O atoms from four carboxyl groups of bpdc anions in an irregular N_2_O_6_ geometry. The carboxylate groups bridge the Pb^II^ cations, forming a three-dimensional polymeric structure. The carboxyl­ate group is twisted away from the attached pyridine ring by 11.4 (3)°.

## Related literature

For background to niacin, see: Krishnamachari (1974 ▶) and to *N*,*N*-diethyl­nicotinamide, see: Bigoli *et al.* (1972 ▶). For related structures, see: Greenaway *et al.* (1984 ▶); Hökelek & Necefoğlu (1996 ▶); Hökelek *et al.* (2009**a* ▶,*b* ▶,*c* ▶,d* ▶, 2010**a* ▶,b* ▶, 2011 ▶).
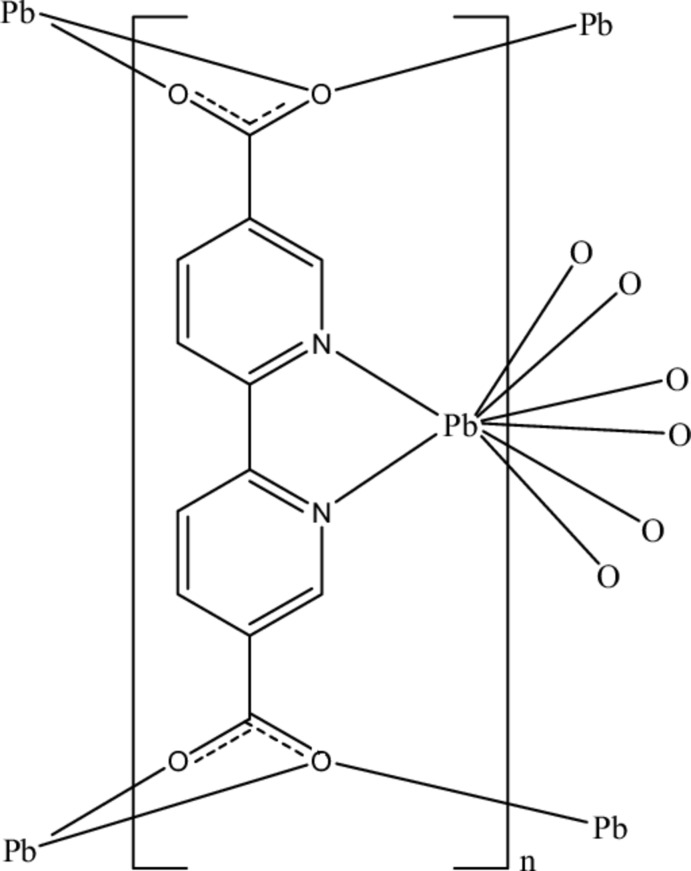


## Experimental

### 

#### Crystal data

[Pb(C_12_H_6_N_2_O_4_)]
*M* *_r_* = 449.39
Orthorhombic, 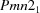

*a* = 13.6224 (3) Å
*b* = 4.1923 (2) Å
*c* = 10.2180 (3) Å
*V* = 583.54 (3) Å^3^
*Z* = 2
Mo *K*α radiation
μ = 14.47 mm^−1^
*T* = 100 K
0.32 × 0.18 × 0.10 mm

#### Data collection

Bruker Kappa APEXII CCD area-detector diffractometer
Absorption correction: multi-scan (*SADABS*; Bruker, 2005 ▶) *T*_min_ = 0.055, *T*_max_ = 0.235
9729 measured reflections
1542 independent reflections
1511 reflections with *I* > 2σ(*I*)
*R*_int_ = 0.036

#### Refinement

*R*[*F* ^2^ > 2σ(*F* ^2^)] = 0.019
*wR*(*F* ^2^) = 0.055
*S* = 1.24
1542 reflections
90 parameters
1 restraint
H-atom parameters constrained
Δρ_max_ = 1.53 e Å^−3^
Δρ_min_ = −0.75 e Å^−3^
Absolute structure: Flack (1983 ▶), 728 Friedel pairs
Flack parameter: 0.497 (14)

### 

Data collection: *APEX2* (Bruker, 2007 ▶); cell refinement: *SAINT* (Bruker, 2007 ▶); data reduction: *SAINT*; program(s) used to solve structure: *SHELXS97* (Sheldrick, 2008 ▶); program(s) used to refine structure: *SHELXL97* (Sheldrick, 2008 ▶); molecular graphics: *ORTEP-3 for Windows* (Farrugia, 1997 ▶); software used to prepare material for publication: *WinGX* publication routines (Farrugia, 1999 ▶) and *PLATON* (Spek, 2009 ▶).

## Supplementary Material

Crystal structure: contains datablock(s) I, global. DOI: 10.1107/S1600536812035647/xu5606sup1.cif

Structure factors: contains datablock(s) I. DOI: 10.1107/S1600536812035647/xu5606Isup2.hkl

Additional supplementary materials:  crystallographic information; 3D view; checkCIF report

## Figures and Tables

**Table 1:** Selected bond lengths (Å)

Pb1—O1^i^	2.819 (5)
Pb1—O2^i^	2.383 (5)
Pb1—O2^ii^	2.860 (5)
Pb1—N1	2.669 (5)

Symmetry codes: (i) 
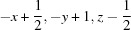
; (ii) 
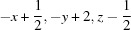
.

**Fig. 1.**
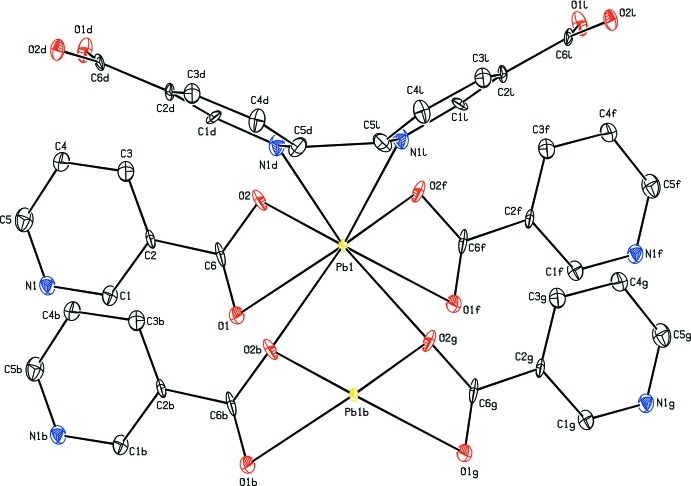
The molecular structure of the title compound with the atom-numbering scheme. Displacement ellipsoids are drawn at the 50% probability level. Atoms are generated by the symmetry operators: (b) x, 1 + y, z, (d) 1/2 - x, 1 - y, 1/2 + z, (f) -x, y, z, (g) -x, 1 + y, z, (l) -1/2 + x, 1 - y, 1/2 + z.

**Fig. 2.**
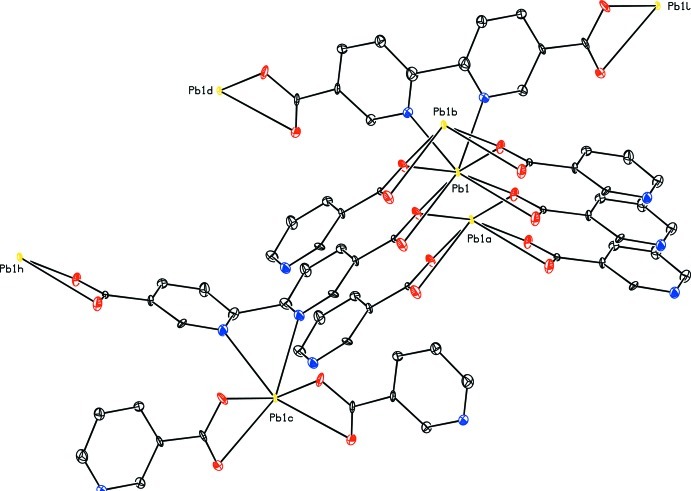
The polymeric structure. Pb atoms are generated by the symmetry operators: (a) x, -1 + y, z, (b) x, 1 + y, z, (c) 1/2 - x, 1 - y, -1/2 + z, (d) 1/2 - x, 1 - y, 1/2 + z, (h) 1 - x, y, z, (l) -1/2 + x, 1 - y, 1/2 + z.

## supplementary crystallographic information

### Comment

As a part of our ongoing investigation on transition metal complexes of nicotinamide (NA), one form of niacin (Krishnamachari, 1974), and/or the nicotinic acid derivative *N*,*N*-diethylnicotinamide (DENA), an important respiratory stimulant (Bigoli *et al.*, 1972) the title compound was synthesized and its crystal structure is reported herein. In fact, in the synthesis we aimed to obtain a mixed complex of lead with nicotinic acid and DENA. But, the nicotinic acid molecules have been interacted to form 2-2'-bipyridine-5-5'-dicarboxylic (bpdc) acid in the hydrothermal synthesis media.

In the crystal structure of the polymeric title compound, (I), the Pb^II^ ion is chelated by the O atoms of the carboxylate groups and the nitrogen atoms from 2-2'-bipyridine-5,5'-dicarboxylato (bpdc)ligands (Fig. 1); the symmetry related Pb^II^ ions are bridged through the O atoms of the carboxyl groups and the nitrogen atoms of the bpdc ligands to form a 3-D polymeric structure (Fig. 2), in which the Pb^II^ ion is in an irregular eight-coordination geometry (Fig. 1).

The Pb–O bond lengths are ranged from 2.383 (5) and 2.860 (5) Å (Table 1) and the Pb atom is displaced out of the least-square plane of the carboxylate group (O1/C6/O2) by -1.5813 (1) Å. The Pb1···Pb1b distance [symmetry code: (b) x, 1 + y, z] is 4.1923 (3) Å (Fig. 1). In (I), the O1–Pb1–O2 and N1–Pb1–N1^i^ [symmetry code: (i) -x, y, z] angles are 50.4 (2) and 52.7 (3) °, respectively.

The corresponding O–M–O (where M is a metal) angles are 51.10 (15)° and 51.95 (16)° in {[Pb(PEB)_2_(NA)].H_2_O}_n_ (Hökelek *et al.*, 2011), 51.09 (6)° and 51.71 (5)° in [Pb(PMB)_2_(NA)]_n_ (Hökelek *et al.*, 2010*a*), 55.96 (4)° and 53.78 (4)° in [Cd_2_(DMAB)_4_(NA)_2_(H_2_O)_2_] (Hökelek *et al.*, 2010*b*), 52.91 (4)° and 53.96 (4)° in [Cd(FB)_2_(INA)_2_(H_2_O)].H_2_O (Hökelek *et al.*, 2009*a*), 60.70 (4)° in [Co(DMAB)_2_(INA)(H_2_O)_2_] (Hökelek *et al.*, 2009*b*), 58.45 (9)° in [Mn(DMAB)_2_(INA)(H_2_O)_2_] (Hökelek *et al.*, 2009*c*), 60.03 (6)° in [Zn(MAB)_2_(INA)_2_].H_2_O (Hökelek *et al.*, 2009*d*), 58.3 (3)° in [Zn_2_(DENA)_2_(HB)_4_].2H_2_O (Hökelek & Necefoğlu, 1996) [where NA, INA, DENA, HB, FB, MAB, PMB, PEB and DMAB are nicotinamide, isonicotinamide, *N*,*N*-diethylnicotinamide, 4-hydroxybenzoate, 4-formylbenzoate, 4-methylaminobenzoate, 4-methylbenzoate, 4-ethylbenzoate and 4-dimethylaminobenzoate, respectively] and 55.2 (1)° in [Cu(Asp)_2_(py)_2_] (where Asp is acetylsalicylate and py is pyridine) (Greenaway *et al.*, 1984).

The dihedral angle between the planar carboxylate group and the adjacent pyridine ring A (N1/C1–C5) is 11.44 (31)°.

### Experimental

The title compound was obtained after leaving a mixture of Pb(NO_3_)_2_ (0.33 g, 1 mmol), nicotinic acid, (NA), (0.24 g, 2 mmol), diethylnicotinamide, (DENA), (0.35 g, 2 mmol) and distilled water (5 ml) in a Teflon-lined autoclave at 433 K for 41 h.

### Refinement

The C-bound H-atoms were positioned geometrically with C—H = 0.95 Å, for aromatic H-atoms, and constrained to ride on their parent atoms, with *U*_iso_(H) = 1.2 × *U*_eq_(C). The highest residual electron density was found 0.89 Å from Pb1 and the deepest hole 0.93 Å from Pb1.

### Crystal data

**Table:**

[Pb(C_12_H_6_N_2_O_4_)]	*F*(000) = 412
*M**_r_* = 449.39	*D*_x_ = 2.558 Mg m^−^^3^
Orthorhombic, *P**m**n*2_1_	Mo *K*α radiation, λ = 0.71073 Å
Hall symbol: P 2ac -2	Cell parameters from 8915 reflections
*a* = 13.6224 (3) Å	θ = 2.5–28.5°
*b* = 4.1923 (2) Å	µ = 14.47 mm^−^^1^
*c* = 10.2180 (3) Å	*T* = 100 K
*V* = 583.54 (3) Å^3^	Prism, colorless
*Z* = 2	0.32 × 0.18 × 0.10 mm

### Data collection

**Table:**

Bruker Kappa APEXII CCD area-detector diffractometer	1542 independent reflections
Radiation source: fine-focus sealed tube	1511 reflections with *I* > 2σ(*I*)
Graphite monochromator	*R*_int_ = 0.036
φ and ω scans	θ_max_ = 28.5°, θ_min_ = 2.5°
Absorption correction: multi-scan (*SADABS*; Bruker, 2005)	*h* = −18→17
*T*_min_ = 0.055, *T*_max_ = 0.235	*k* = −5→5
9729 measured reflections	*l* = −13→13

### Refinement

**Table:**

Refinement on *F*^2^	Hydrogen site location: inferred from neighbouring sites
Least-squares matrix: full	H-atom parameters constrained
*R*[*F*^2^ > 2σ(*F*^2^)] = 0.019	*w* = 1/[σ^2^(*F*_o_^2^) + (0.0212*P*)^2^ + 3.443*P*] where *P* = (*F*_o_^2^ + 2*F*_c_^2^)/3
*wR*(*F*^2^) = 0.055	(Δ/σ)_max_ < 0.001
*S* = 1.24	Δρ_max_ = 1.53 e Å^−^^3^
1542 reflections	Δρ_min_ = −0.75 e Å^−^^3^
90 parameters	Extinction correction: *SHELXL*, Fc^*^=kFc[1+0.001xFc^2^λ^3^/sin(2θ)]^-1/4^
1 restraint	Extinction coefficient: 0.0097 (6)
Primary atom site location: structure-invariant direct methods	Absolute structure: Flack (1983), 728 Friedel pairs
Secondary atom site location: difference Fourier map	Flack parameter: 0.497 (14)

### Special details

**Table:**

Geometry. All esds (except the esd in the dihedral angle between two l.s. planes) are estimated using the full covariance matrix. The cell esds are taken into account individually in the estimation of esds in distances, angles and torsion angles; correlations between esds in cell parameters are only used when they are defined by crystal symmetry. An approximate (isotropic) treatment of cell esds is used for estimating esds involving l.s. planes.
Refinement. Refinement of F^2^ against ALL reflections. The weighted R-factor wR and goodness of fit S are based on F^2^, conventional R-factors R are based on F, with F set to zero for negative F^2^. The threshold expression of F^2^ > 2sigma(F^2^) is used only for calculating R-factors(gt) etc. and is not relevant to the choice of reflections for refinement. R-factors based on F^2^ are statistically about twice as large as those based on F, and R- factors based on ALL data will be even larger.

### Fractional atomic coordinates and isotropic or equivalent isotropic displacement parameters (Å^2^)

**Table:**

	*x*	*y*	*z*	*U*_iso_*/*U*_eq_	
Pb1	0.0000	0.81379 (5)	0.6243	0.00544 (10)	
O1	0.3476 (4)	0.2412 (13)	0.9391 (5)	0.0145 (9)	
O2	0.3886 (3)	0.6151 (12)	1.0873 (4)	0.0136 (9)	
N1	0.0870 (4)	0.7262 (14)	0.8556 (5)	0.0134 (11)	
C1	0.1730 (4)	0.5765 (15)	0.8787 (5)	0.0099 (11)	
H1	0.1929	0.4075	0.8227	0.012*	
C2	0.2338 (4)	0.6648 (14)	0.9835 (6)	0.0091 (11)	
C3	0.2044 (5)	0.9052 (17)	1.0680 (6)	0.0138 (11)	
H3	0.2446	0.9677	1.1395	0.017*	
C4	0.1157 (5)	1.0506 (17)	1.0457 (6)	0.0149 (12)	
H4	0.0926	1.2117	1.1034	0.018*	
C5	0.0596 (5)	0.9597 (17)	0.9373 (6)	0.0160 (12)	
C6	0.3296 (4)	0.4896 (16)	1.0016 (5)	0.0127 (12)	

### Atomic displacement parameters (Å^2^)

**Table:**

	*U*^11^	*U*^22^	*U*^33^	*U*^12^	*U*^13^	*U*^23^
Pb1	0.00259 (13)	0.00704 (13)	0.00668 (12)	0.000	0.000	0.00030 (19)
O1	0.009 (2)	0.022 (2)	0.013 (2)	0.0027 (19)	−0.0030 (18)	−0.0058 (18)
O2	0.007 (2)	0.018 (2)	0.015 (2)	−0.0035 (17)	−0.0056 (13)	−0.0002 (15)
N1	0.009 (3)	0.022 (3)	0.009 (2)	0.000 (2)	−0.001 (2)	−0.002 (2)
C1	0.009 (3)	0.013 (3)	0.008 (2)	0.004 (2)	−0.0023 (19)	0.004 (2)
C2	0.003 (3)	0.014 (3)	0.011 (3)	−0.001 (2)	−0.002 (2)	0.0008 (19)
C3	0.010 (3)	0.025 (3)	0.006 (2)	0.001 (3)	0.000 (2)	0.000 (3)
C4	0.010 (3)	0.018 (3)	0.017 (3)	0.003 (2)	−0.001 (2)	−0.004 (2)
C5	0.012 (3)	0.020 (3)	0.016 (3)	0.005 (2)	−0.002 (2)	0.004 (2)
C6	0.003 (2)	0.029 (4)	0.006 (2)	−0.004 (2)	−0.0017 (18)	0.006 (2)

### Geometric parameters (Å, º)

**Table:**

Pb1—O1^i^	2.819 (5)	C1—C2	1.403 (8)
Pb1—O2^i^	2.383 (5)	C1—H1	0.9500
Pb1—O2^ii^	2.383 (5)	C2—C3	1.386 (9)
Pb1—O2^iii^	2.860 (5)	C2—C6	1.509 (8)
Pb1—N1	2.669 (5)	C3—C4	1.373 (9)
Pb1—N1^iv^	2.669 (5)	C3—H3	0.9500
O1—C6	1.245 (8)	C4—C5	1.398 (9)
O2—Pb1^v^	2.383 (5)	C4—H4	0.9500
N1—C1	1.350 (8)	C5—C5^iv^	1.623 (13)
N1—C5	1.340 (9)	C6—O2	1.300 (7)
			
O2^ii^—Pb1—O2^i^	79.1 (2)	C3—C2—C1	119.8 (6)
O2^i^—Pb1—N1^iv^	108.61 (16)	C3—C2—C6	121.8 (5)
O2^ii^—Pb1—N1	108.61 (17)	C2—C3—H3	120.9
O2^i^—Pb1—N1	75.73 (16)	C4—C3—C2	118.3 (6)
O2^ii^—Pb1—N1^iv^	75.73 (16)	C4—C3—H3	120.9
N1—Pb1—N1^iv^	52.7 (3)	C3—C4—C5	119.4 (6)
C6—O2—Pb1^v^	101.2 (4)	C3—C4—H4	120.3
C1—N1—Pb1	127.2 (4)	C5—C4—H4	120.3
C5—N1—Pb1	109.1 (4)	N1—C5—C4	122.7 (6)
C5—N1—C1	118.2 (6)	N1—C5—C5^iv^	106.2 (4)
N1—C1—C2	121.5 (6)	C4—C5—C5^iv^	123.1 (4)
N1—C1—H1	119.2	O1—C6—O2	124.2 (6)
C2—C1—H1	119.2	O1—C6—C2	120.9 (5)
C1—C2—C6	118.4 (5)	O2—C6—C2	114.8 (6)
			
O2^i^—Pb1—N1—C1	19.7 (5)	N1—C1—C2—C6	−179.0 (5)
O2^ii^—Pb1—N1—C1	92.8 (5)	C1—C2—C3—C4	−0.1 (10)
O2^i^—Pb1—N1—C5	172.7 (5)	C6—C2—C3—C4	−179.3 (6)
O2^ii^—Pb1—N1—C5	−114.2 (5)	C1—C2—C6—O1	−11.3 (9)
N1^iv^—Pb1—N1—C1	147.1 (5)	C1—C2—C6—O2	169.9 (5)
N1^iv^—Pb1—N1—C5	−59.9 (5)	C3—C2—C6—O1	167.9 (6)
Pb1—N1—C1—C2	149.5 (5)	C3—C2—C6—O2	−10.9 (8)
C5—N1—C1—C2	−1.3 (9)	C2—C3—C4—C5	−1.9 (10)
Pb1—N1—C5—C4	−156.5 (5)	C3—C4—C5—N1	2.5 (11)
Pb1—N1—C5—C5^iv^	53.8 (3)	C3—C4—C5—C5^iv^	147.0 (5)
C1—N1—C5—C4	−0.8 (10)	O1—C6—O2—Pb1^v^	−14.7 (7)
C1—N1—C5—C5^iv^	−150.5 (5)	C2—C6—O2—Pb1^v^	164.0 (4)
N1—C1—C2—C3	1.8 (9)		

Symmetry codes: (i) −*x*+1/2, −*y*+1, *z*−1/2; (ii) *x*−1/2, −*y*+1, *z*−1/2; (iii) −*x*+1/2, −*y*+2, *z*−1/2; (iv) −*x*, *y*, *z*; (v) −*x*+1/2, −*y*+1, *z*+1/2.
